# Sustained Release of an Anti-Glaucoma Drug: Demonstration of Efficacy of a Liposomal Formulation in the Rabbit Eye

**DOI:** 10.1371/journal.pone.0024513

**Published:** 2011-09-09

**Authors:** Jayaganesh V. Natarajan, Sujay Chattopadhyay, Marcus Ang, Anastasia Darwitan, Selin Foo, Ma Zhen, Magdalene Koo, Tina T. Wong, Subbu S. Venkatraman

**Affiliations:** 1 School of Materials Science and Engineering, Nanyang Technological University, Singapore, Singapore; 2 Department of Chemical Engineering, Doctor Babasaheb Ambedkar Technological University, Lonere, Maharashtra, India; 3 Singapore National Eye Centre, Singapore, Singapore; 4 Ocular Drug Delivery Group, Singapore Eye Research Institute, Singapore, Singapore; Institute Biomedical Research August Pi Sunyer (IDIBAPS) - Hospital Clinic of Barcelona, Spain

## Abstract

Topical medication remains the first line treatment of glaucoma; however, sustained ocular drug delivery via topical administration is difficult to achieve. Most drugs have poor penetration due to the multiple physiological barriers of the eye and are rapidly cleared if applied topically. Currently, daily topical administration for lowering the intra-ocular pressure (IOP), has many limitations, such as poor patient compliance and ocular allergy from repeated drug administration. Poor compliance leads to suboptimal control of IOP and disease progression with eventual blindness. The delivery of drugs in a sustained manner could provide the patient with a more attractive alternative by providing optimal therapeutic dosing, with minimal local toxicity and inconvenience. To investigate this, we incorporated latanoprost into LUVs (large unilamellar vesicles) derived from the liposome of DPPC (di-palmitoyl-phosphatidyl-choline) by the film hydration technique. Relatively high amounts of drug could be incorporated into this vesicle, and the drug resides predominantly in the bilayer. Vesicle stability monitored by size measurement and DSC (differential scanning calorimetry) analysis showed that formulations with a drug/lipid mole ratio of about 10% have good physical stability during storage and release. This formulation demonstrated sustained release of latanoprost in vitro, and then tested for efficacy in 23 rabbits. Subconjunctival injection and topical eye drop administration of the latanoprost/liposomal formulation were compared with conventional daily administration of latanoprost eye drops. The IOP lowering effect with a single subconjunctival injection was shown to be sustained for up to 50 days, and the extent of IOP lowering was comparable to daily eye drop administration. Toxicity and localized inflammation were not observed in any treatment groups. We believe that this is the first demonstration, in vivo, of sustained delivery to the anterior segment of the eye that is safe and efficacious for 50 days.

## Introduction

Glaucoma is the second leading cause of blindness in the world [1]. Intraocular pressure (IOP) is the main, modifiable risk factor for this disease. Drugs to treat IOP are classified by the mode of action of the active ingredient eg. prostaglandin analogs, beta blockers, alpha agonists and carbonic anhydrase inhibitors [2]. In addition, fixed combination drugs are now available for patients who require more than one type of medication. Currently, medications used to lower IOP are applied topically, which have poor ocular bioavailability, side effects associated with chronic use (allergic conjunctivitis and dry eyes) and require patient reliance on daily administration [3]–[8]. Since glaucoma requires life-long treatment, drug delivery for glaucoma remains a challenging problem, as currently only eye drops are available for topical drug delivery which is associated with very variable therapeutic efficacy and also heavily dependent on patient compliance.

One of the main barriers to ocular drug delivery is the corneal epithelium. It is a tri-lamellate structure consisting of a hydrophilic rigid stromal layer of cells sandwiched between two lipophilic (epithelium and endothelium) layer of cells [9], [10]. Of the two common pathways (paracellular and transcellular) that have been proposed for the transport of drug molecules, the drug molecules would predominantly use the transcellular path to cross the cornea, where the lipophilicity and pKa are the major parameters that decide the entry of drug molecules [9]. However, in addition to the permeation obstacles, the tear drainage also causes any topically applied drug to get washed away fairly rapidly. Because of all these factors, a mere 5% of the free drug applied on the corneal epithelium successfully penetrates through the cornea. In order to improve bioavailability of drug, the transport barrier as well as prolonging the retention of the drug carrier in the anterior segment of the eye needs to be achieved.

Latanoprost, a lipophilic drug molecule is usually effective for reducing the IOP. Latanoprost is an isopropyl ester of its corresponding acid, which is the active constituent. In the eye, it is expected that the ester is hydrolyzed into IPA (isopropyl alcohol) and latanoprost acid [11]. The latanoprost acid has an aqueous solubility of 5 mg/ml [12]. However, the latanoprost acid experiences a higher penetration resistance through the lipophilic epithelium and the endothelial cells of the corneal membrane. Latanoprost is usually delivered in the form of either an oil/water emulsion (Xalatan®), or lipid/buffer emulsion [11], [13]. There is another unavoidable issue of irreversible yellow pigmentation of corneal epithelium after regular application of commercial eye drop for longer period (beyond three months). This pigmentation is attributed to the presence of benzalkonium chloride (which acts as a preservative for latanoprost in this formulation).

Therefore, to circumvent these critical issues of (i) frequent instillations for required efficacy, (ii) drug stability/clearance and (iii) undesirable side effects of drug, several delivery vehicles have been studied in the literature [14]. Of the carriers proposed, liposomes were thought to be the most promising [15]–[17], to overcome these challenges for higher therapeutic efficacy and sustained release.

Liposomes are versatile vehicles for incorporation of both hydrophilic and lipophilic drug molecules, due to its physical structure with a polar core and a lipophilic bilayer. Liposomal encapsulation also protects drug molecules from enzymatic hydrolysis in the physiological environment. However, the size of small uni-lamellar vesicle (SUV, 20–50 nm) or large uni-lamellar vesicle (LUV, ∼100 nm) often restricts its transport through epithelial layers, and also permits rapid clearance during topical administration. Thus, several modifications of liposomes have been reported: (i) surface modification with charged lipids which can make vesicles adhere to the oppositely (negatively) charged corneal epithelium, (ii) increasing the lipophilicity of the drug molecules, and (iii) modifying the integrity of corneal epithelium transiently by using a penetration enhancer. Such modifications would facilitate the first stage of the entry of drug molecules, while the subsequent permeation would be governed by pKa, hydrophilicity/lipophilicity of the drug molecules [10], [18].

A review report by Meisner and Mezei [19] compared various routes of administration (e.g. topical administration, intravitreal, subconjunctival and systemic injection) of liposomal formulations. The first successful application of topically applied drug-loaded liposome (MLVs) formulation was carried out in the rabbit eye by Smolin *et al* [20]. In a separate study, Schaeffer and Krohn [21] have shown that the order of liposomal uptake by the corneal epithelium varied as follows: MLV+ (MLVs with positively charged lipid)>SUV+ (SUVs with positively charged lipid)≫MLV− (MLVs with negatively charged lipid>SUV− (SUVs with negatively charged lipids)>MLV = SUV. However, the overall permeation of a water soluble drug, penicillin G, was highest for SUV+ and was minimal for MLVs and free drugs, due to the large surface area of SUVs. Delivery of a hydrophobic drug due to its low water solubility limit is considerably more challenging. The limited bilayer space availability in the lipid bilayer often limits the loading of a lipophilic drug and is the most significant hurdle in developing liposomes for sustained delivery of hydrophobic entities, including latanoprost.

Lin *et al* [22] found that in vitro application of norfloxacin loaded liposome onto cornea showed improved drug retention when compared to free drug solution. They indicated a probable corneal endocytic uptake of norfloxacin loaded liposome through the corneal membrane. There appears to be no other report of enhanced drug retention in the anterior segment of the eye. The pharmacokinetics of drug release from the liposome vesicle and penetration through corneal epithelium is still poorly understood. If the drug release from these vehicles is appreciably slow, then clearance will dominate drug adsorption and diffusion through corneal epithelium [23]. Therefore, released drug will be too diluted (tear dilution) to have an adequate gradient to cross the corneal epithelium [24]. Thus, it may perform poorer than even free drug.

There are very few successful in vivo studies reported for topically applied liposome loaded drug formulations. Hathout *et al* have shown that positively charged egg phosphatidyl choline (PC): cholesterol (CH): stearyl amine (7∶4∶1) multilamellar vesicles (MLVs) loaded with acetazolamide, when applied topically, shows the highest lowering of IOP (−7.8 mm Hg) after 3 hrs. However this lowering lasts only for about 8 hours [25]. Hirnle *et al* have compared sub-conjunctival delivery of 6-carboxyfluorescein (as a “model” drug) in rabbit eyes in aqueous solution and liposomal formulation. The released drug concentration was noticeably high after 30 min and transitory conjunctivitis (common swelling due to excess drug concentration) was observed, but no side effect was reported. The liposomal formulation showed detectable levels of carboxyfluorescein at the injection site of sub-conjunctiva along with sclera, cornea, choroid and retina after 7 days of injection, but the reason for the 7 day sustainability/retention in this study was not clearly understood [26].

Although liposomal carriers have been evaluated for delivery of drugs topically on the front of the eye, none have been successful to date for sustained release. Very few studies have been reported on the fate of liposomes injected subconjunctivally. Nevertheless, subconjunctival injection may be an attractive option for sustained delivery of anti-glaucoma agents, provided the effects can be sustained for at least 1 month, preferably longer. Specifically, for the delivery of latanoprost (a prostaglandin derivative that is currently administered via once-a-day eye drops), there is no report on comparison of in vitro and in vivo release behavior. In this work, we have evaluated liposomal formulations based on dipalmitoylphosphatidylcholine (DPPC). DPPC lipids have saturated acyl chains and are expected to remain stable in vivo. In the present report, we have evaluated carrier stability, drug partitioning, in vitro drug release, toxicity, efficacy and sustainability of latanoprost-incorporated DPPC liposomes upon subconjunctival administration in rabbit eyes.

## Materials and Methods

### 1.1. Materials

DPPC was purchased from NOF Corporation, Japan. Cellulose ester dialysis bags (16 mm dia×10 m flat width) were obtained from Spectrum labs, USA. Polycarbonate filter membranes of sizes (0.2, 0.1 and 0.08 µm) and drain discs were purchased from Northern Lipids Inc, Canada. Chemicals for phospholipid analysis such as ammonium thiocyanate and ferric chloride hexahydrate were purchased from Sigma, USA. Sodium phosphate dibasic anhydrous (Na_2_HPO_4_), Potassium phosphate monobasic anhydrous (KH_2_PO_4_), Potassium chloride (KCl), Sodium chloride (NaCl) salts were purchased from Sigma, USA and used without further purification. Latanoprost was purchased from Chemical Testing and Calibration Laboratories, China. The water used in all the experiments was from MiliQ purification system with a resistivity of at least 18.2±0.2 mΩ-cm. All solvents for drug estimation was carried out using high performance liquid chromatograph (HPLC) grade (>99% purity) and were purchased from Tedia, USA.

### 1.2. Preparation of Large Unilamellar Vesicles (LUVs)

Latanoprost loaded liposomes were formulated by thin film hydration technique as described elsewhere [27]. Briefly, known amount of DPPC (lipid concentration, 15 mM) was weighed and dissolved in chloroform: methanol (2∶1, v/v) solvent mixture. A known amount of drug dissolved in acetonitrile (ACN) was added to this mixture and maintained in a 40°C water bath. The drug**∶**lipid mole ratios were varied between 0.04 to 0.24. Solvents were removed from the round bottom flask using a rotary evaporator (IKA RV 10, Germany) maintained in a 40°C water bath (IKA MB 10 basic, Germany). A thin dry drug loaded lipid film was obtained and to this film, isotonic PBS (150 mM, pH 5.5) buffer was added and sonicated for 3–5 min, to form multilamellar vesicles (MLVs). We avoided a longer exposure time to ultrasound, due to possibilities of increased lipid oxidation and/or drug leakages as reported elsewhere [28]. After the completion of the sonication step, the resulting formulations were extruded 10–15 times sequentially through polycarbonate filters of size (0.2 µm/0.1 µm/0.08 µm), fitted in a bench top extruder (Northern Lipids Inc, Canada). This will result in formation of LUVs with a size distribution of 0.09–0.12 µm. All the above-mentioned steps were performed under aseptic conditions. All glassware, PBS (filtered through 0.2 µm filter) solution were sterilized by autoclaving, and the entire procedure was performed under a laminar flow hood (Esco, Singapore). Sterility (Method suitability test and Sterility test) conditions of the drug loaded formulations were verified through an external agency (BRASS, Biomedical Research and Support Service Pvt. Ltd., Singapore) and found to meet the desired criteria.

### 1.3. In Vivo Study in Rabbits

#### 1.3.1. Topical application of liposomal formulation as eye drop

Six female New Zealand white rabbits were divided into two treatment groups. Daily IOP values were monitored and a stable IOP baseline value was observed by the end of seven days. One treatment group (3 rabbits, 6 eyes) received one daily drop of liposomal formulation and the other treatment group received a drop of Xalatan® formulation (3 rabbits, 6 eyes).

#### 1.3.2. Subconjunctival delivery of latanoprost loaded liposomes

Twenty three female New Zealand White rabbits (both eyes, BE = 46 eyes) were used in this study. The baseline IOP was measured twice daily using a calibrated Tono-pen XL for 7 days in all rabbits. The rabbits were divided into 3 treatment groups: (i) Group A (8 rabbits, 16 eyes) received 1 eye drop of topical latanoprost daily; (ii) group B (8 rabbits, 16 eyes) received 1 subconjunctival injection of latanoprost-loaded liposomes and (iii) group C (7 rabbits, 14 eyes) received subconjunctival injection of liposomes only (with no drug). Rabbit eyes in groups B and C received a subconjunctival injection of liposomes at day 0 and at day 50 using 27 gauge sterile needles, since the IOP returned to the baseline values. All procedures were performed under topical anesthesia (amethocaine 2.5%) by a single surgeon (MA). In brief, the eye was cleaned with 50% povidone iodine and 0.1 ml of liposome was injected in the subconjunctival space in the superior temporal region of each eye. Topical chloramphenicol 2.5% was administered to the operated eye daily for 5 days. We obtained approval from the SingHealth Institute Animal Care and Use Committee and all procedures were performed in accordance with the ARVO Statement for the Use of Animals in Ophthalmic and Vision Research.

#### 1.3.3. Clinical evaluation

Twice-daily IOP measurements were conducted using a calibrated Tono-pen XL and visual inspection of all eyes for clinical signs of inflammation in the eye and at the injection site and for any evidence of infection. Slit-lamp examination of the exterior and anterior chamber of the eyes was done prior to injections and weekly thereafter. Anterior segment photographs and anterior segment optical coherence tomography (AS-OCT) of the operated eyes was performed at weekly intervals. The rabbits were also monitored for any gross changes such as eye discharge, squinting or abnormal behavior. We used a modified version of the Moorfields bleb grading system to objectively assess vascularity in all eyes [29].

#### 1.3.4. Histology

At the end of the study period (80 days) all rabbits were sacrificed and sampled aqueous and vitreous humor for latanoprost concentrations via HPLC analysis. Euthanasia was carried out with intraperitoneal pentobarbitone (60–150 mg/kg) followed by enucleation of the operated eyes. Sterile syringes with 27-gauge needles were used to sample 0.1 ml of aqueous humor and 0.1 ml of vitreous humor before immediately immersing the eyes in a mixture of 4% paraformaldehyde and 2.5% neutral buffered formalin for 24 hours. The globes were dehydrated, embedded in paraffin and sent for microtome sectioning and respective staining (Haematoxylin and Eosin (H&E) and Sirius red F3BA; Sigma, St. Louis, MO). Polarization microscopy of stained collagen fibers for any gross collagen bundling patterns and a modified semi-quantitative grading system as previously described was used to assess fibrosis and scarring [30].

#### 1.3.5. Statistical analysis

Statistical analysis included descriptive statistics, where the mean and standard deviation (SD) was calculated for the continuous variables; while frequency distribution and percentages were used for categorical variables. Comparisons between categorical variables were conducted by Fisher's exact tests, whereas the one-way analysis of variance (ANOVA) test was used for means. A P-value<0.05 was considered statistically significant.

### 1.4 Characterization of drug loaded liposome vesicles

#### 1.4.1. Size and Zeta Potential

The average size of the liposomes as well as the size distribution (polydispersity index, PDI) of each formulation was estimated using the Malvern Zetasizer Nano ZS (UK). A small aliquot of the formulation was diluted with a large (1∶100, v/v) volume of distilled water and any air drop interference while measurement was eliminated all throughout the measurement. Vesicle sizes were monitored following synthesis, on storage at 4°C and after drug release. The zeta potential values were estimated by Laser Doppler method using the same instrument in 0.001 M PBS buffer at 25°C. DPPC lipids was ensured that more than 75% of latanoprost was entrapped into the liposome while measurement. As indicated in Table 1, there was no appreciable change in particle size, zeta potential, although entrapment efficiency was noted to be slightly higher with lower drug/lipid mole ratio.

**Table 1:** Size, Zeta potential and loading efficiency of latanoprost loaded DPPC Liposome after extrusion.

DPPC Liposomes(D/L, mole/mole)	Size(nm)	PDI	Zeta potential(mV)	Drug Loading Efficiency(%)
0.04	90±10	0.09–0.12	−4.6±2.0	85±3%
0.104	120±20	0.1–0.2	−7.1±2.2	80±5%

#### 1.4.2. Lipid Analysis

The phospholipid concentration was estimated using colorimetric method as described elsewhere [31]. Six point calibration was prepared for DPPC (ëmax = 525 nm) and were later used for estimation of unknown concentration of lipids in vesicles obtained after extrusion. The amount of phospholipids in liposomal vesicles after extrusion was estimated to be 80–85% (by mass) while, the remaining was considered lost while extrusion and handling.

#### 1.4.3. DSC Analysis of the Drug and DPPC interaction

DSC analysis of the latanoprost loaded DPPC liposome formulations was done using TA instruments Q10 model. The instrument Q10 model was calibrated with Indium for the best Cp estimate and high signal to noise ratio. Number of heating/cooling cycles, heating rate, volume of liposome formulation, and concentration in DI water/PBS buffer were all established by comparing the standard DSC chromatograms available for DPPC and its variation with cholesterol mole % [32]. The DSC chromatograms obtained from Q10 undoubtedly needed higher lipid mass compared to nano DSC, but the nature of DSC chromatograms were same, and therefore was chosen for further data interpretation. A heating rate of 1°C/min between temperature ranges −20°C to 60°C was selected to avoid any thermal decomposition of drug molecule [33]. A third heating cycle was always considered for all DSC analysis. The results were reproducible for 100 mg/ml liposomal formulations and a sample volume of 10 ul was taken in a hermitic pan for analysis. An empty pan was used as a reference.

#### 1.4.4. Drug concentration using High Performance Liquid Chromatography Method

The HPLC system (Agilent series 1200) consisted of solvent degasser, high precision pump, auto sampler, column heater, column, and a photo diode (UV) array detector. Each unit was interfaced with computer through Chemstation software. The chromatographic separation was performed on reverse phase Eclipse-XDB C_18_ column (5 µm, 4.6 mm ID×250 mm) using mobile phase as ACN∶Water at 70∶30 (v/v) proportions at 1.0 ml/min flow rate and detector wavelength was set at ë = 210 nm. The retention time was 4.6 min and the temperature of the column was maintained at 25°C.

The drug estimation from release medium was done directly from the collected samples. While, the drug present in the lipid vesicles needed a separate treatment. Lipid vesicles of drug loaded formulation were broken by adding 1∶4 volumes of IPA (isopropyl alcohol) and the lipid mass was isolated from the rest of solution by ultracentrifugation at 13000 rpm for 30 minutes. The supernatant was diluted 50× times with PBS buffer to match within the calibration limit. HPLC system was calibrated (R^2^ = 0.998) using known concentrations of latanoprost solution (25, 10, 7.5, 5, 2.5, 1.0, 0.1 µg/ml) by diluting latanoprost dissolved in ACN in PBS buffer (150 mM, pH 7.4). The estimation of the total drug was carried out by breaking the liposomal vesicles with IPA. The broken liposomal mixture was centrifuged and the clear supernatant was diluted with PBS buffer and estimated by HPLC. Each sample was tested at least five times to obtain concordant data of the total drug concentration of stock liposomal formulations. Amount (%) of drug released at various time were plotted against time.
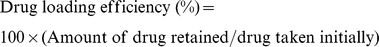



#### 1.4.5. Drug partition coefficient estimation

A lipophilic drug such as latanoprost distributes between a lipid bilayer and the aqueous continuous phase and the concentration ratio between these two phases determines the partition coefficient. The values for partition coefficient were estimated from the MLVs prior to the extrusion step. Briefly, known sample volumes were collected in microfuge tubes and centrifuged at 10000 rpm for 30 mins. Due to the large size of MLVs, in the micron size ranges, we were able to separate out the fractions of the lipid pellet and the clear supernatant. The drug estimated from the supernatant is a measure of continuous (buffer) phase drug concentration, while this amount when subtracted from the total drug concentration yields drug partitioned into the bilayer. Thus, drug partition coefficient (P.C.) is estimated using the following expression (each concentration in mass per unit volume):
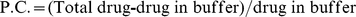



Drug: Lipid ratio (D/L) is an important characteristic property of liposomal systems. The final drug: lipid mole ratios in the liposomal formulation obtained after extrusion were estimated as follows:

Final drug to lipid (D/L) mole ratio = Moles of drug left/Moles of the lipid left after extrusion.

#### 1.4.6. Drug Release studies

In this study, the drug loaded liposomal formulation was physically separated from the receptor chamber by a dialysis membrane and the released drug amounts were assayed from the release medium at various time intervals. Briefly, 1 ml of drug loaded liposomal suspension was taken in a cellulose ester dialysis bag (100 kD MWCO, 1.6 cm dia×6 cm length) and clipped at both ends using dialysis clips. The whole assembly was then placed in screw capped bottle (to avoid evaporation loss during the whole duration of release study) containing 40 ml PBS buffer (150 mM, pH 7.4: 137 mM NaCl, 2.68 mM KCl, 1.76 mM KH_2_PO_4_, 10.14 mM Na_2_HPO_4_). A volume ratio of 1∶40 (liposome∶buffer) was chosen to maintain a pseudo-sink condition for drug release. The dialysis bag was continuously agitated in an orbital shaker (Sartorius Cartomat, USA) maintained at 37°C at 50 rpm. Aliquots were withdrawn from the release medium and was filtered through 0.2 µm syringe filter and collected in amber color HPLC sample bottle (2 ml, Agilent) followed by storage in the fridge at 4°C until they were estimated. To obtain a closer approximation to the dynamic sink condition in the in-vitro setup, the dialysate was completely exchanged with fresh PBS buffer after every 24 h and the released drug concentration in the dialysate was estimated as discussed before. Each experiment was repeated at least 3 times. The volume of liposomal formulation and the sizes of vesicle in the dialysis tube were recorded at the end of the release study to check any dilution effect of liposomal formulation and size changes of vesicles that occurred during drug release.

## Results

### 2.1. Drug loading Efficiency

The observed encapsulation efficiency was in the range 75–88% for various formulations as reported in Table 1. The concentration of loaded drug after extrusion was estimated to be 200–660 ug/ml which is 4–12 fold higher than the Xalatan® (50 ug/ml) eye drop formulation whose normal dose requirement is one drop a day [34]. The drug to lipid mole ratio (Table 2) after extrusion is higher than the initial value, which indicates that there is more partial loss (∼20%) of lipid molecules than of drug molecules in the various stages of size reduction. These mole ratios are much higher than the values obtained for a comparable lipophilic drug, ibuprofen, in the cholesterol-containing lipid vesicle of Mohammed *et al* [35]. The estimated mole ratios of drug/lipid were in the range between 0.04–0.37 (Table 2). The highest drug/lipid mole ratio we obtained was nearly 3–5 times higher than earlier reported ibuprofen loading by Mohammed *et al* [35] or Cisplatin by Perez-Soler and Khokhar [36]. However, this very high loading of latanoprost did not affect much on the vesicle stability (LUVs).

**Table 2:** Drug loading values before and after synthesis of liposome vesicles.

Drug: Lipid mole ratio		
Initial	Final	Literature
0.030	0.044±0.001	0.02 with ibuprofen in DPPC: Chol [35]
0.065	0.073±0.002	----
0.086	0.104±0.020	----
0.114	0.140±0.020	----
0.143	0.180±0.030	----
0.170	0.370±0.050	0.29 with aryl-imidazole (ML220) in DSPC-PEG [40]

### 2.2. In vitro size stability

**Figure 1:**
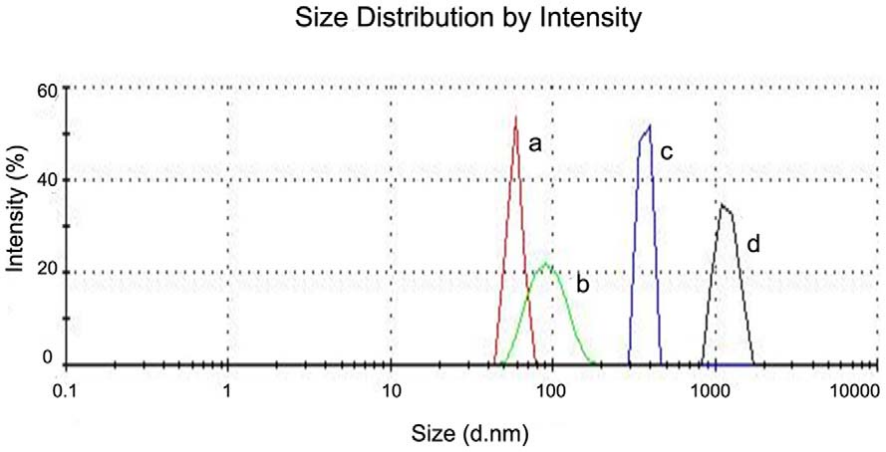
Malvern Zeta sizer analysis of drug loaded liposome. The values reported are after 12 weeks of storage at 4°C with drug/lipid mole ratio (size, nm) (a) 0.044 (63±10), (b) 0.11 (90±30), (c) 0.18 (200±80) and (d) 0.37 (1250±200). Size measurement reported here are mean of three batches and the standard deviations (±) are expressed inside bracket.

The size measurements at various time intervals eg. W0 (initial), W2 (two weeks later), W4 (four weeks later) were carried out using the Zetasizer. From Table 3, it is seen that vesicles are reasonably stable while stored at 4°C as long as the drug/lipid ratio is low. Effect of vesicle destabilization was noted when the drug/lipid mole ratio exceeded 0.1. This was expected since the high loading would disrupt the structure of the bilayer, with shape distortion, as reported from another lipophilic drug in a similar vesicle [37]. Figure 1 shows the sizes after 12 weeks of storage of drug-loaded vesicles at 4°C, and it is evident that the larger loading leads to greater size changes (all the vesicles start out at roughly the same size).

**Table 3:** Size measurement of liposome formulation (varying d/l mole ratio) during storage at 4°C and after in vitro drug release in PBS buffer (pH 7.4) at 37°C.

Size measurement, nm (PDI)						
Latanoprost/DPPC Liposome (actual) drug/lipid mole ratio)	W0	W2	W4	W8	W16	After in vitro drug release
0.00	82(0.05)	93(0.06)	----	91(0.06)	92(0.06)	93(0.06)
0.04	86(0.05)	89	102(0.06)	104(0.06)	104(0.06)	110(0.06)
0.07	111(0.07)	89(0.06)	98(0.06)	100(0.06)	----	----
0.10	109(0.08)	105(0.10)	105(0.10)	----	110(0.10)	150(0.1)
0.14	78(0.06)	93(0.06)	93(0.08)	101(0.08)	117(0.10)	145(0.12)
0.18	194(0.10)	210(0.10)	210(0.10)	—	310(0.20)	442(0.20)
0.37	118(0.10)	93(0.10)	93(0.20)	1015(0.3)	1176(0.30)	2020(0.80)

### 2.3. Drug release behavior

**Figure 2:**
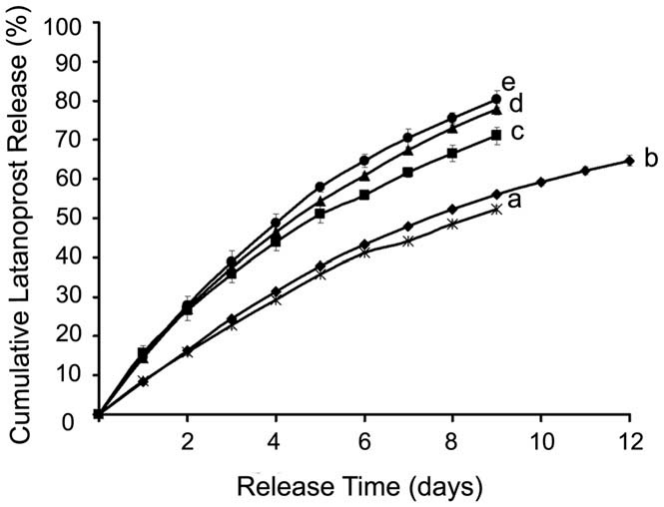
In vitro dialysis study of latanoprost from drug loaded DPPC LUVs after extrusion. Cumulative latanoprost release (%) from DPPC LUVs loaded with varying amount of drug/lipid mole ratios: (a) 0.044, (b) 0.11, (c) 0.14, (d) 0.18 and (e) 0.37. Each value is the mean (standard deviations is plotted as error bars, which are always less than 3% for nearly all batches) of the results obtained from at least three independent experiments.

Latanoprost release behavior from loaded liposomal formulations varying in drug/lipid mole ratio, is expressed in terms of cumulative drug release (%) (Figure 2). In order to better mimic the in vivo release condition, the buffer solution was exchanged every 24 h with fresh PBS pH 7.4 maintained at 37°C. The release rate of any formulation increases with the initial drug concentration. There is no appreciable change between drug/lipid mole ratios 0.04 to 0.11, but, beyond this limit the rate increases.

For a better understanding of the release pattern, vesicle sizes were recorded at the end of every release study (Table 3). For molar ratios lesser than drug/lipid mole ratio of 0.14, the relative size variation is negligibly small; however, with higher amounts of drug, the size variations and polydispersity were higher, indicating probable vesicle destabilization during release, thus leading to faster drug release. As discussed in the stability section, the higher drug concentrations in the bilayer might affect the vesicle stability and thus accelerate drug release. Further understanding comes from estimation of thermal stability of drug loaded DPPC lipid bilayer using differential scanning calorimetry (DSC) measurements.

### 2.4. DSC analysis

**Figure 3:**
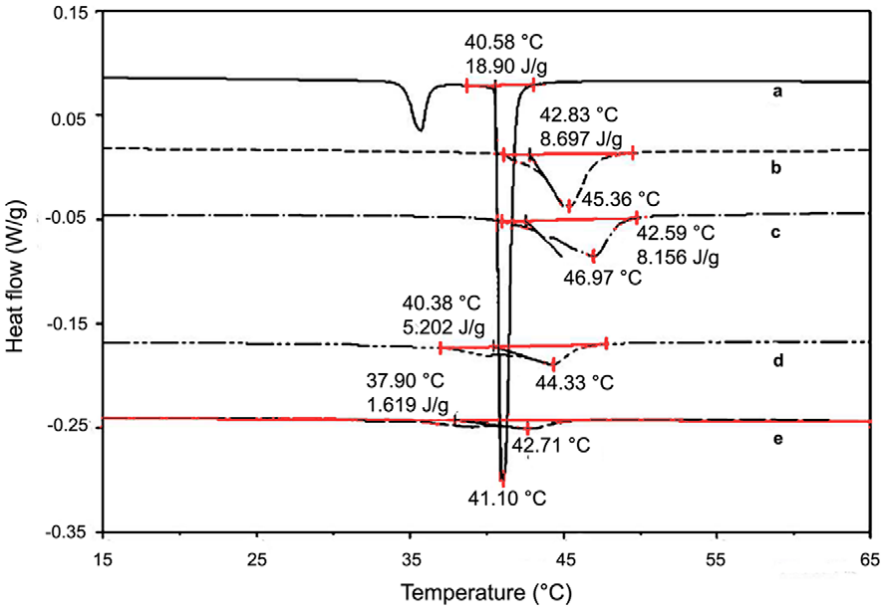
DSC analysis of pure and latanoprost loaded DPPC MLVs. The liposome was made by PBS buffer hydration of anhydrous drug-lipid layer. 10 µl of the liposomal formulation (100 mg/ml) was heated at 1°C/min between −20°C to 65°C. Three heating and two cooling cycles were carried out with each sample and the last reproducible heating cycle was considered for analysis. The drug/lipid mole ratios and their corresponding enthalpy change during transition are reported within parentheses (a) 0.0 (18.9 J/g), (b) 0.03 (8.7 J/g), (c) 0.086 (8.1 J/g), (d) 0.114 (5.2 J/g) and (e) 0.14 (1.6 J/g).

Figure 3 shows the DSC thermograms of different latanoprost loaded DPPC liposome. The DSC shows endothermic transition for both drug-free and drug-loaded liposome. Plain DPPC liposome shows a sharp main transition (41.1°C) with an expected pre transition at (35°C) as well. As the drug/lipid mole ratio is increased, the pre transition disappears and the endothermic main transition broadens and shifts (Figure 3). It is also interesting to note the shift is towards a higher temperature as the drug/lipid mole ratio changes from 0 to 0.086 (41.1 to 47°C). The trend then reverses and shifts towards lower temperature, although with a considerable peak broadening. With higher drug amount (drug/lipid mole ratio of 0.114) the transition disappears completely. This observed effect could be an indication of the gradual change from more ordered liquid to more disordered liquid behavior, caused by excessive drug incorporation. The peak broadening with latanoprost incorporation resembles the trend reported for cholesterol in DPPC lipid bilayer [38].

The lipophilic latanoprost molecules would preferentially locate themselves in the lipid bilayer, and thus might influence the stability of the bilayer. Excess drug loading may affect stability of vesicles, which could yield a “leakier” liposome and thus faster release.

### 2.5. Partition coefficient of latanoprost in DPPC liposome

**Figure 4:**
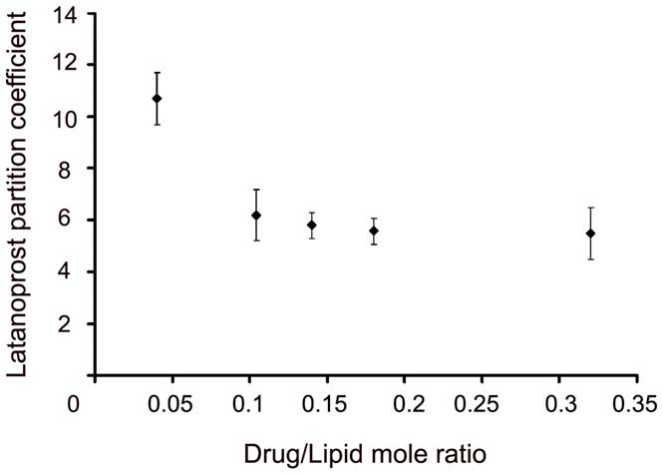
Partition coefficient of latanoprost. Partition coefficient of latanoprost was calculated by taking the ratio of drug concentrations in lipid bilayer and the aqueous buffer. The drugs to lipid loading concentrations (mole ratios) are 0.04, 0.104, 0.14, 0.18, and 0.32 in DPPC. Each partition coefficient value was obtained from the mean of three MLV formulations made and the standard deviations are reported in error bars.

The ratio of drug concentration in the lipid bilayer to that in the continuous phase is defined as partition coefficient (P.C.), as discussed earlier. As shown in Figure 4, a decrease in partition coefficient from 11.0±1 to 6.0±1 was observed upon varying the drug/lipid mole ratios from 0.04 to 0.104. However, with any further increase in the drug/lipid mole ratios up to 0.32, no differences in the partition coefficient were observed.

### 2.6. Selection of DPPC liposome formulation for in vivo studies in rabbit eyes

The data is based on the vesicle storage stability, DSC analysis, drug partition coefficient and daily dose requirement. A liposomal formulation of drug/lipid mole ratio of 0.104 was chosen to study the IOP lowering effect in the rabbit eye, since the current eye drop administration of Xalatan® amounts approximately to 1.5 µg/drop/day [39].

**Figure 5:**
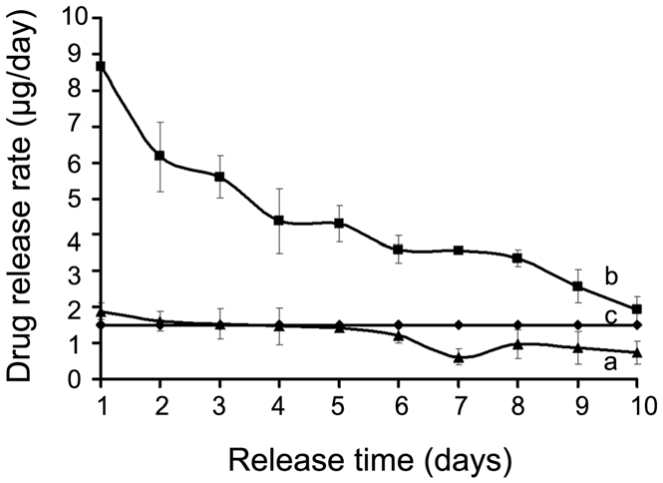
Latanoprost release rate from 100 µl of DPPC LUVs with two different drug/lipid mole ratios compared with 1 drop of commercial Xalatan® solution (1.5 µg/drop). In vitro drug release rate (µg/day) measured from (a) drug/lipid, 0.04 and (b) drug/lipid, 0.11, (c) commercial eye drop (Xalatan®, 1.5 µg/drop). The release rates are reported based on mean values of at least two batches and standard deviations are reported in error bars.

In the sub-conjunctival administration of the liposomal latanoprost, we wanted to target a similar dose/day sustained for at least 10 days, assuming a subconjunctival injection volume of 100 µL. The in vitro release rate (per day) is plotted for two candidate formulations of DPPC-latanoprost, at drug/lipid mole ratios of 0.04 and 0.104, in Figure 5 shown for comparison with the daily dose of Xalatan® eye drop. Although the drug/lipid mole ratio of 0.04 matches the eye drop, its daily rate dips below Xalatan® beyond Day 5, and hence, is unsuitable for sustained delivery. Thus, we opted to use the DPPC-latanoprost formulation at a drug/lipid mole ratio of 0.104 for the rabbit study, even though it demonstrated a higher initial burst release in vitro.

### 2.7. Topically applied liposomal formulation

We also considered topical administration for the DPPC-latanoprost formulation, mimicking the eye drop administration of Xalatan®. However, IOP measurement in all the rabbits did not show any IOP reduction (data not reported) compared to the Xalatan® eye drop. This shows that latanoprost loaded neutral DPPC liposomal formulation once applied topically cannot enter through corneal epithelium. This mode of administration of the liposomal formulation was not considered further and only the sub-conjunctival injection was compared to the eye drop in the rabbit studies.

### 2.8. Effects of intraocular pressure from subconjunctival injection of latanoprost/liposomal formulation

**Figure 6:**
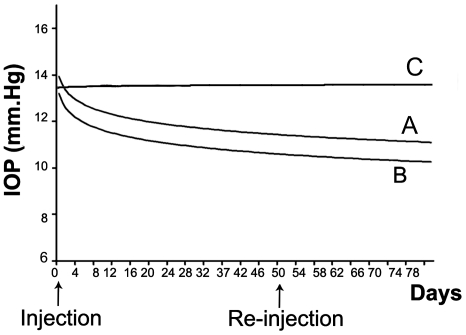
Comparison of intraocular pressure (IOP) between topical latanoprost eyedrop, subconjunctival latanoprost liposome and latanoprost-free blank liposomes. Group A: topical latanoprost eye drop. Group B: subconjunctival latanoprost liposome. Group C: latanoprost-free blank liposomes.

The baseline IOP was 13.6±0.3 mmHg for all 23 rabbits and there was no significant difference between all 3 groups (P = 0.81) evaluated. We found a significantly higher mean reduction of IOP in the subconjunctival latanoprost-loaded liposome (group B) compared to topical latanoprost eye drop (group A). At 30 days IOP reduced by 3.0±0.17 mmHg in group B versus 1.6±0.18 mmHg in group A (P<0.001) and at 80 days the IOP reduced by 3.5±0.60 vs. 2.3±0.61 mmHg respectively (P<0.001) (Figure 6).

### 2.9. Subconjunctival injection of latanoprost/liposome formulation

**Figure 7:**
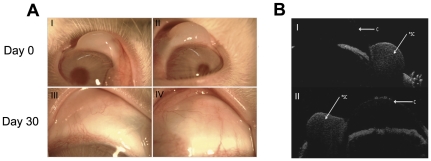
Rabbit eyes condition after subconjunctival injection. Serial slit-lamp microscopy (group A) revealed no significant increase in vascularity and inflammation of all rabbit eyes (I and II: Day 0 after subconjunctival injection; III and IV: Day 30 after subconjunctival injection). AS-OCT scans (group B) revealed no abnormal scarring, scleral or conjunctival thinning in all rabbit eyes (Photos I and II). *SC = Liposome injection site; C = Cornea.

We recorded the clinical effect of injecting the liposome formulation on conjunctival vascularity shortly after administering the subconjunctival injection and at Day 30 in each treatment group. No significant increase in conjunctival vascularity in any of the eyes from the 3 groups was noted. Furthermore, we did not observe any complications such as conjunctival infection, ulceration or anterior chamber inflammation on slit-lamp examination (Figure 7A). The surrounding conjunctiva was white and the eyelids did not show any signs of swelling or inflammation. Finally, ASOCT scans confirmed the subconjunctival placement of the injected liposomes in the superior temporal aspect of each rabbit eye (Figure 7B). Subsequent ASOCT scans did not reveal any abnormal conjunctival scarring or scleral thickening at the injection sites, thus supporting the slit lamp observations.

### 2.10. Histology

**Figure 8:**
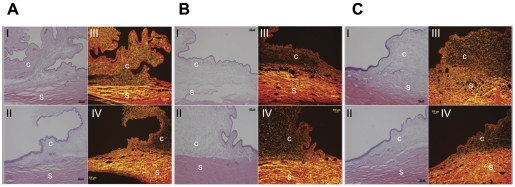
Histology of rabbit eyes. Group A: topical latanoprost eyedrops. Group B: subconjunctival latanoprost liposomes. Group C: subconjunctival latanoprost-free liposomes. Histology revealed no abnormal scarring or damage to the collagen layers in both H&E stain (Photos I and II) and Picrosirius red stain (Photos III and IV; Grade 1 for all eyes). S = Sclera; C = Conjunctiva.

The surrounding conjunctival tissue did not reveal any significant inflammation or fibrosis on histological examination of all the enucleated eyes. Sirius red polarizing microscopy of collagen fibers also revealed no significant increase in the amount of fibrosis in all 3 groups (Figure 8).

## Discussion

It is now recognized that there is a medical need for sustained delivery of eye drops needed for chronic diseases such as glaucoma. We selected one such agent, a prostaglandin derivative, latanoprost for incorporation into liposomes for sustained delivery. We were able to achieve high (75–88%) drug loading into the DPPC liposomes. The lipophilic nature of latanoprost with a logP = 4.4 (Data from SRC PhysProp Database) clearly indicates that the loaded drug resides predominantly in the bilayer. However, a measurable amount of latanoprost also resides in the aqueous phase outside of the liposomes. This is reflected in the measured partition coefficients, which vary from about 12 to 6, depending on initial drug loading. These numbers translate to about 8% and 17% “free drug” outside of the liposomes. Free drug removal from the formulation was not carried out, as removal requires a fairly lengthy process. Removal of free lipophilic drug (ibuprofen) from liposomal formulation is also not routinely performed [35]. Therefore, all the experiments and analyses were based on the formulation obtained after extrusion step, which contains mostly liposome-incorporated latanoprost, with about 17% free drug in the case of the 0.104 drug/lipid mole ratio formulation.

The drug/lipid mole ratio estimated here is nearly 3–5 times more than earlier reported lipophilic drugs eg. Ibuprofen [35] and Cisplatin [36]. A similar higher loading of lipophilic anti-cancer drug aryl- imidazole compound (ML220) loaded in DSPE-PEG liposome have a reported 3 weeks of stability at the incubation temperature (37°C) [40].

Storage stability analysis shows that vesicles with no drug or with lower drug amounts (drug/lipid mole ratio of 0.14) remain unaffected. When the quantity of drug is increased to drug/lipid mole ratio of 0.18 and above, destabilization is noted from 4 weeks onwards. Size analysis after drug release at 37°C in PBS buffer shows similar behavior to that during storage. The destabilization is magnified at the higher drug/lipid formulations. A possible explanation for the destabilization of the vesicles with increase in loading lies in the interactions of latanoprost with the bilayer acyl/phosphate head group of DPPC liposomes. Latanoprost molecules (at high loading) could possibly weaken the hydrogen bonding between the phosphate head groups that hold vesicle together [41]. However; this postulate remains to be confirmed with suitable studies. Thus, vesicles with a lesser amount of drug (till drug/lipid mole ratio of 0.14) are stable for at least one month.

In general, we noted drug release rate to increase with an increase in drug/lipid mole ratios. There are currently no accepted models for the release of drugs from liposomes. If we treat the liposomal carriers as “monolithic matrices” and assume mainly diffusional release of drug, then for either dissolved or dispersed drug, we expect a decrease in rate with loading rather than an increase [42]. However, we observed no dependence on loading when the drug/lipid ratios ranged from 0.04 to 0.11, but an increase in rate with loading for drug/lipid mole ratios >0.11 was seen. This increase is attributable to a more “destabilized” vesicle in terms of size increase during the release study (Table 3), which implies the creation of a larger vesicle bilayer more permeable to diffusion. Beyond drug/lipid ratios of 0.11, the latanoprost appears to be loaded beyond its saturation limit in the bilayer, and as a consequence, the metastable bilayer changes in size over time and being destabilized.

The thermal stability (DSC) analysis of drug loaded DPPC liposome indicates peak broadening with drug loading. The sharp transitions (pre- and main) disappear (Figure 3) with drug incorporation. Mannock *et al* [38] reported peak broadening due to incorporation of cholesterol in DPPC bilayer and attributed this to probable loss of crystalline nature of DPPC lipids. However, unlike cholesterol incorporation into DPPC, which leads to a drop in transition temperature, latanoprost loaded DPPC liposomes behave differently. At the release condition, the bilayer is expected to remain in the rigid gel phase (high crystalline) for DPPC (Tc = 41.1°C). With lower amount of drug/lipid mole ratio there is an increase in transition temperature from 41.1 for drug/lipid = 0 to 44.8 for drug/lipid = 0.04 and 46.6 with drug/lipid = 0.086. Recently, Bolean *et al* [43] have reported a similar peak broadening and rise in transition temperature with higher incorporation of cholesterol in DPPC liposome. At high molar concentrations, cholesterol exists as a sterol rich domain (also called “non-cooperative zone”) leading to peak broadening and a shift towards higher temperature [44]. This domain formation is also reflected in an increase in vesicle diameter which is similar to our observation with higher mole % of latanoprost in DPPC (Table 3). DPPC lipid volume was estimated [45] to be ∼1200 Å^3^/molecule (of which ∼900 Å^3^ is for acyl chains and rest ∼300 Å^3^ for phosphate head group) while the latanoprost molecule has an approximate volume of ∼500 Å^3^ based on molecular dynamic calculations [46]. With increased proportion of latanoprost, gradually the lipid-latanoprost interaction increases due to bilayer space constraint, which may lead to an increase in transition temperature of the binary system [43]. However, the existence of a maximum in the transition temperature (with drug/lipid mole ratio) requires further investigation. The enthalpy change (Figure 3) at the main transition with pure DPPC (18.9 J/g) gradually decreases to 8.7, 8.1, 5.2, 1.6 J/g respectively with increase in drug/lipid mole ratios at 0.03(b), 0.086 (c), 0.114 (d), 0.14 (e). This also supports the postulated latanoprost-lipid interaction that renders the bilayer more fluidic or disordered [44].

Partition coefficient analysis indicates a drop in drug distribution between the lipid bilayer and the continuous phase, with increased drug loading. After the initial decrease (from about 11 to about 6), the partition coefficient remains unaltered. The partition coefficient gives an estimate of the drug distribution between the lipid bilayer and the continuous phase. The method of lipophilic drug incorporation also strongly influences the partition coefficient value [47].

For smaller drug quantities such as drug/lipid mole ratio of 0.04, the drug molecules prefer to remain in the bilayer as entrapped drug and the rest as free drug in the buffer. The accumulation of the drug in the bilayer is limited by the bilayer area available in the vesicles. Favorable drug-bilayer interactions could increase this bilayer loading as well. For higher drug loading amounts, there is a significant amount of the drug existing as free drug in the continuous buffer phase of the liposomes, as the bilayer gets saturated with drug molecules. This appears to explain the decrease seen in the partition coefficient values seen in our study

In vivo studies revealed that a single depot subconjunctival injection of latanoprost-loaded liposome results in a sustained, therapeutic lowering of IOP beyond 50 days. The extent of IOP lowering was significantly better using the liposomal formulation than free latanoprost administered daily via eye drop, the current ‘standard of treatment’ in glaucoma patients. This is consistent with our in vitro results that show a sustained release (and a higher amount released compared to the eye drop) over 10 days, while maintaining vesicle size and shape. The significant and sustained reduction of IOP in rabbit eyes is encouraging, and to our knowledge, there are no other reports that show such a dramatic improvement in latanoprost sustainability by DPPC liposomes via subconjunctival injections in an animal eye. We also used slit-lamp microscopy, histology and imaging techniques such as AS-OCT to demonstrate that these liposomes appear to be well tolerated in an animal eye, biocompatible and does not cause any obvious clinical adverse effects to the ocular tissues with the subconjunctival injection of the liposomal carrier.

Because of the measured higher initial rate of drug release from the DPPC formulation, there were concerns about side effects during initial stages following injection. However, throughout the duration of our study, we did not detect any obvious effect with the methods used for clinical monitoring. There was also no residual drug either in the aqueous or vitreous humor at the end of study. It is also interesting to note that repeat injections of drug loaded DPPC liposome treatment did not result in any hyperpigmentation to the cornea or conjunctiva. Unlike the commercial eye drop (Xalatan®), the present formulation is devoid of any preservatives such as benzalkonium chloride (BAK), which is thought to be the root cause of the cornea pigmentation [48]. The short period of exposure to the drug may also be a reason for the absence of clinical corneal changes.

### 3.1. Conclusions

The IOP lowering effect from a single subconjunctival injection of the reported liposome formulation was sustained for up to 50 days and was comparable to daily administration of eye drops. The outcomes from our studies report positive and significant benefits over the commercial eye drop formulation. Subconjunctival injection of liposomes to provide sustained delivery of ocular drugs may prove to be a suitably attractive alternative to current topical eyedrops.
